# Significant Expression Levels of Transgenic PPP1CC2 in Testis and Sperm Are Required to Overcome the Male Infertility Phenotype of *Ppp1cc* Null Mice

**DOI:** 10.1371/journal.pone.0047623

**Published:** 2012-10-17

**Authors:** Nilam Sinha, Stephen Pilder, Srinivasan Vijayaraghavan

**Affiliations:** 1 Department of Biological Sciences, Kent State University, Kent, Ohio, United States of America; 2 Department of Anatomy and Cell Biology, Temple University School of Medicine, Philadelphia, Pennsylvania, United States of America; The Walter and Eliza Hall of Medical Research, Australia

## Abstract

PPP1CC2, one of four isoforms of the ser/thr protein phosphatase PP1, is a mammalian-specific splice variant of the *Ppp1cc* gene, and the only isoform whose expression is confined almost completely to spermatogenic cells. Additionally, PPP1CC2 is the sole isoform found in mammalian spermatozoa. Although PPP1CC1, the other *Ppp1cc* product, is expressed in many tissues including testis, the only phenotype resulting from deletion of *Ppp1cc* gene is male infertility. To determine which of the products of *Ppp1cc* is essential for male fertility, we created two PPP1CC2 transgenes, eTg-G2 and pTg-G2, where *Ppp1cc2* expression was driven by the putative endogenous promoter of *Ppp1cc* or by the testis specific human *Pgk2* promoter, respectively. Our results demonstrate that the 2.6-kb genomic region directly upstream of the *Ppp1cc* structural gene can drive expression of *Ppp1cc2,* and recapitulate the wild-type tissue specificity of PPP1CC2 in transgenic mice. More importantly, we show that expression of PPP1CC2 alone, via either promoter, is able not only to restore normal spermatogenesis, but the fertility of *Ppp1cc* null mice as well, provided that transgenic PPP1CC2 expression in testis reaches at least a lower threshold level equivalent to approximately 50% of its expression by a *Ppp1cc +/−* male. We conclude that the endogenous *Ppp1cc* promoter normally functions in the testis to maintain a sufficient level of PPP1CC2 expression for normal spermatogenesis to occur, and that production of spermatozoa capable of fertilization in vivo can take place in the complete absence of PPP1CC1 expression.

## Introduction

Throughout the reproductive life span of the male, complex signaling mechanisms govern each step of spermatogenesis, resulting in the continuous production of morphologically mature testicular spermatozoa and their release from their association with Sertoli cells into the lumen of the seminiferous tubule [1], [2]. However, the testicular spermatozoa of mammals do not become motile or acquire their ability to bind and fertilize eggs until they undergo a maturation process in the epididymis [3], [4]. Just as spatial and temporal variations in protein phosphorylation patterns mediate many of the processes of sperm development in the testis, so do they control sperm motility initiation in the epididymis, and regulation of mature sperm function following ejaculation. In all cases, these changes are regulated by the combined activities of protein kinases and protein phosphatases.

Type 1 serine/threonine protein phosphatases (PP1 isoforms) belong to the PPP (phospho-protein phosphatase) gene family. PP1 protein phosphatases are present in all eukaryotic organisms ranging from budding yeast to mammals. They play key roles in glycogen metabolism, muscle contraction, and cell division [5], [6], [7]. Four protein isoforms of PP1 (PPP1CA, PPP1CB, PPP1CC1, and PPP1CC2) are ultimately derived from three genes in mammals (*Ppp1ca*, *Ppp1cb*, *Ppp1cc*). *Ppp1cc1* and *Ppp1cc2* are differentially spliced products of the *Ppp1cc* gene [8], an event that occurs only in mammalian species. While all isoforms other than PPP1CC2 are expressed in a wide range of tissues [9], PPP1CC2 is predominantly expressed in testis where it is restricted to meiotic and post-meiotic germ cells [10], [11], [12].

The PP1 isoforms share a high degree of amino acid sequence similarity (∼ 90%), and are, thus, among the most evolutionarily conserved proteins known [13], [14]. This high level of conservation allows any of the mammalian PP1 isoforms to complement yeast (*S. cerevisiae*) lacking its single native PP1 (Glc7p) [15]. The differences between the primary sequences of mammalian PP1 isoforms reside mostly in their extreme C-termini, the functions of which have not yet been completely elucidated [16]. PPP1CC2, with its unique 22 amino acid COOH-tail [8], [17], is present only in mammals. Remarkably, the C-terminus of PPP1CC2 is virtually unchanged in all mammals for which annotated genomic databases exist (www.ensembl.org). This high degree of conservation suggests a role for the C-terminus in interactions specific to PPP1CC2.

Previous studies have revealed that PPP1CC2 plays a key role in sperm motility development [18], [19]. High protein phosphatase activity correlates with sperm immotility in the caput and corpus epididymis. Inhibition of PP1 catalytic activity results in motility initiation in immotile sperm, and increases in kinetic activity of motile bovine and monkey epididymal and human ejaculated spermatozoa [19], [20], [21]. Additionally, its presence in the sperm head as well as the tail suggests that PPP1CC2 may regulate other sperm functions important for fertilization [22]. Interestingly, targeted deletion of the *Ppp1cc* gene (*Ppp1cc* −/−), eliminating both *Ppp1cc1* and *Ppp1cc2* isoforms, results only in male infertility due to highly impaired spermatid morphogenesis and an inability to spermiate, so that the epididymides of *Ppp1cc* −/− mice are virtually devoid of spermatozoa [12], [23]. In contrast, *Ppp1cc −/−* female mice are fertile and appear normal in all other respects, suggesting that PPP1CA and/or PPP1CB can substitute for the loss of the PPP1CC isoforms in all tissues except testis. These findings have indicated an indispensible role for PPP1CC1 and/or PPP1CC2 in spermatogenesis and male fertility.

To determine if one or both PPP1CC isoforms is/are essential for spermatogenesis and male fertility, we initially attempted to rescue the *Ppp1cc −/−* phenotype by expressing transgenic rat *Ppp1cc2* driven by the testicular germ cell specific human *Pgk2* promoter (*hPgk2*) in the *Ppp1cc −/−* genetic background [24]. The results of these early experiments demonstrated that even relatively low levels of PPP1CC2 expression in the testis of *Ppp1cc −/−* males could re-establish spermatid viability and release of testicular spermatozoa into the lumen of the seminiferous tubule, but could not restore fertility. The mature spermatozoa isolated from the caudae epididymides of rescue males demonstrated poor forward motility at best, most likely due to the wide range of morphological abnormalities, found primarily, but not exclusively, in their flagella. We speculated that four primary reasons for the lack of complete rescue were: (1) the relatively low levels of transgenic PPP1CC2 expression in rescue mice; (2) the fact that the *Pgk2* promoter cannot recapitulate precisely the endogenous spatio-temporal expression pattern of PPP1CC2 in testis; (3) PPP1CC1 expression in spermatogonia and Sertoli cells is also required; or (4) any combination of 1, 2, and/or 3.

To distinguish between these possibilities, we have created a new mouse *Ppp1cc2* cDNA transgene driven by the endogenous promoter of *Ppp1cc*, and added new transgenic mouse lines to those in which rat *Ppp1cc2* expression was driven by the human *Pgk2* promoter, although we have substituted mouse *Ppp1cc2* cDNA for rat cDNA. Our current results reveal that, regardless of the promoter employed for transgene expression, restoration of normal spermatogenesis and fertility in *Ppp1cc −/−* males is critically dependent on levels of PPP1CC2 expression in both developing testicular germ cells and spermatozoa equal to or greater than 50% of the levels expressed by a *Ppp1cc +/−* male. As in our previous experiments [24], lower expression in the testis results in a high percentage of structurally defective sperm and infertility, although profound testicular apoptosis and the complete absence of spermiation observed in *Ppp1cc* null males are overcome, even in the poorest PPP1CC2 expressing lines. Thus, as steady state levels of PPP1CC2 expression rise, the number of structurally normal, motile spermatozoa isolated from the caudae epididymides and vas deferens increases until fertility is restored, regardless of the promoter used to express *Ppp1cc2*. This study demonstrates that a lower threshold level of PPP1CC2 is able to restore fertility to a *Ppp1cc −/−* male in the complete absence of PPP1CC1 expression.

## Results

### Generation of *Ppp1cc2* Transgenic Rescue (*Ppp1cc* −/−; *Tg*+) Lines

**Figure 1:**
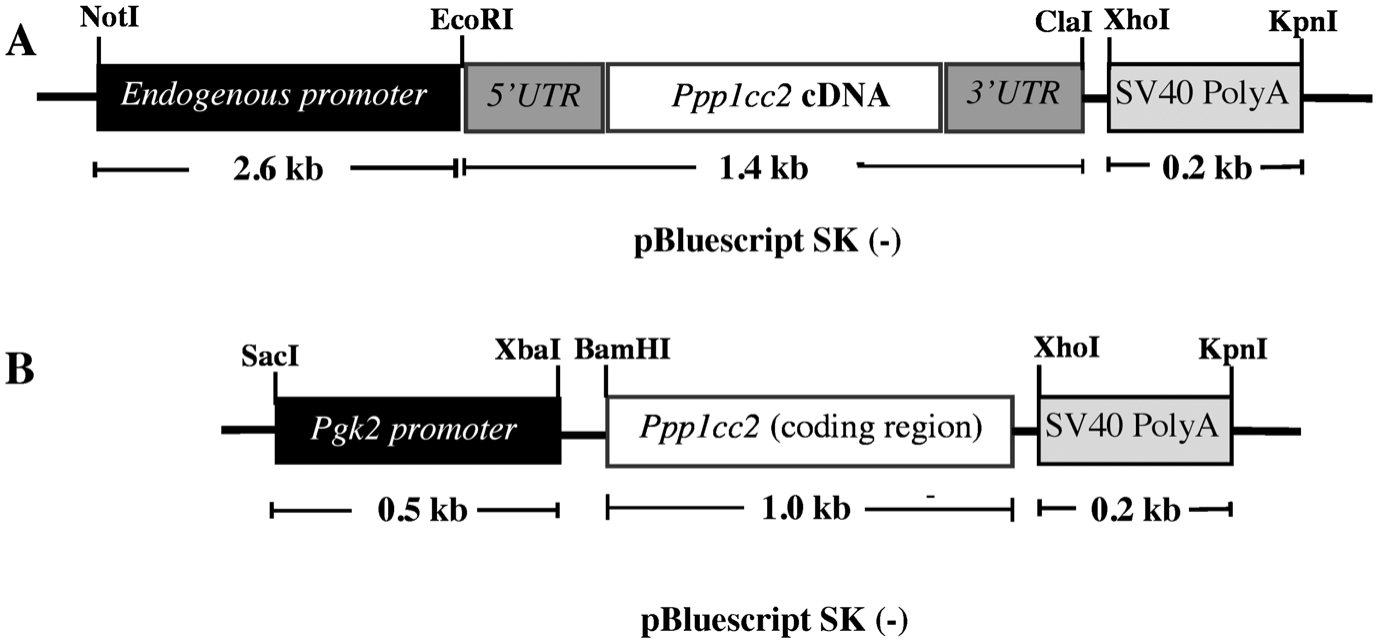
Design of transgenic rescue constructs. (**A**) Transgene mini cassette construct where transgene expression was driven by the putative endogenous promoter fragment of *Ppp1cc* gene. A genomic fragment of ∼2.6 kb immediately upstream of the transcription start site was employed as the endogenous promoter to drive the expression of full length mouse *Ppp1cc2* cDNA (with intact 5′UTR and 3′UTR). (**B**) Testis specific human *Pgk2* promoter driven transgene cassette. In this mini gene the mouse *Ppp1cc2* coding sequence used lacked the 5′ and 3′UTRs. In both constructs the SV40 PolyA signal sequence was added 3′ of the cDNA construct.

Because our earlier attempts to rescue the *Ppp1cc −/−* infertility phenotype failed [24], we developed mouse lines where transcription of transgenic mouse *Ppp1cc2* cDNA was driven by the endogenous 2.6-kb region immediately upstream of the transcription start site of the *Ppp1cc* gene (hereafter called the endogenous *Ppp1cc* promoter) (Fig. 1A). As a matter of promoter comparison, we also added additional transgenic lines to those in which rat *Ppp1cc2* transcription had been driven by the *hPgk2* promoter [25]. However, although mouse and rat PPP1CC2 proteins are identical, small differences in their mRNAs exist at the nucleotide level. Thus, in our new *hPgk2* promoter construct, the mouse *Ppp1cc2* coding region was substituted for the rat *Ppp1cc2* coding sequence (Fig. 1B).

Four of 33 offspring resulting from microinjection of the endogenous promoter construct were transgene positive. These animals were crossed to CD1 *Ppp1cc* −/− females to derive founder lines eTg-M7; eTg-F1; eTg-F3; eTg-F10 (where eTg denotes the endogenous promoter driven transgene, and the alphanumeric number following it denotes the sex and serial number of the founder animal, respectively). The F1 males (*Ppp1cc +/−*; Tg+) from the founder lines were further crossed to CD1 *Ppp1cc* −/− females to derive *Ppp1cc* transgenic rescue males (*Ppp1cc−/−*; Tg+). Three lines were established (eTg-M7; eTg-F1; eTg-F10) with confirmed transgenic protein expression. Microinjection of the new *hPgk2* transgene construct yielded seven transgene positive founders of which we were able to establish three transgenic rescue lines (pTg-M3, pTg-M26 and pTg-M30). Transgene transmission in both the endogenous and *hPgk2* promoter driven *Ppp1cc2* rescue lines followed expected Mendelian ratios.

### Expression of Transgenic PPP1CC2 via Either Promoter Restores Spermatogenesis and Spermiation to *Ppp1cc−/−* Males

**Figure 2:**
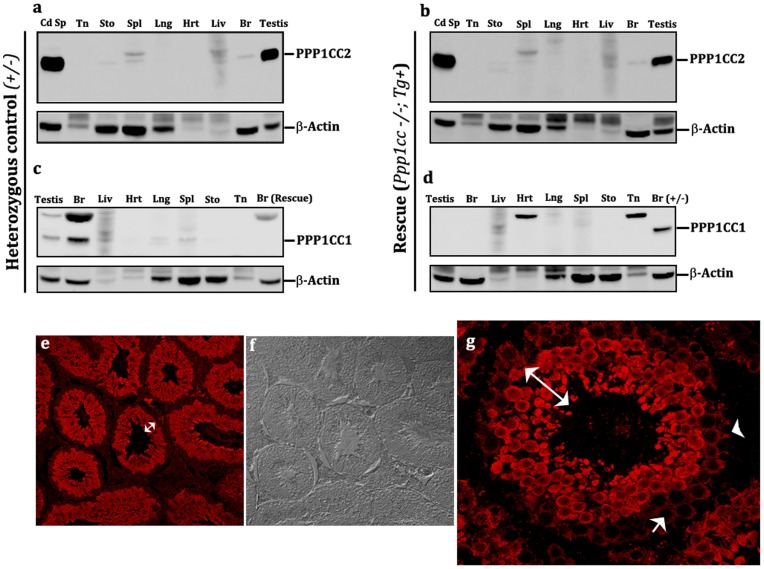
Testis specificity of the 2.6 kb endogenous promoter fragment. (**a, b**) Tissue western blot showing the testis specificity of the *Ppp1cc* gene promoter. PPP1CC2 expression is detected in high amounts in testis and relatively low amounts in brain (Br), spleen (Spl), liver (Liv) in rescue males, comparable to the positive control pattern of expression. No PPP1CC2 reactivity was observed in other somatic tissues studied, including tongue (Tn), stomach (Sto), lungs (Lng) and heart (Hrt). (**c, d**) Multi-tissue western blot analysis of PPP1CC expression in rescue lines vs. control animals. The absence of PPP1CC1 expression in the testis and brain confirms the *Ppp1cc* null background of the rescue mice in comparison to control mice where the isoform is expressed. (**e, f, g**) Immunohistochemistry of testis sections from endogenous promoter- transgenic rescue lines (eTg), showing a wild-type pattern of PPP1CC2 localization in the testis. Double headed (white) arrows indicate PPP1CC2 expression in developing germ cells. Arrow heads points towards its absence in sertoli cells, spermatoginia, pre-leptotene and leptotene spermatocytes. Images were obtained at 20×(e and f) and 60×(g) using confocal fluorescent microscopy. These images are representative observations taken from multiple sections of all three eTg lines (M7, F1 and F10) and several different testis preparations.

Expression patterns of PPP1CC2 in all three pTg rescue lines tested were restricted to late meiotic and post-meiotic germ cells of the testis, as expected from our previous study [24]. Additionally, western blot analysis of different tissue extracts and immuno-histochemical (IHC) evaluation of testis sections showed that PPP1CC2 expression patterns in all three eTg-transgenic rescue lines were similar to those in wild-type mice: PPP1CC2 expression was predominantly testicular with small amounts detected in brain, spleen, liver and stomach extracts (Fig. 2a, b). Also as expected, PPP1CC2 immuno-reactivity was not detected in heart, lungs and tongue. Spatio-temporal expression of transgenic PPP1CC2, as determined by IHC analysis, was also indistinguishable from that of wild-type testis (Fig. 2e, f, g) [12]: transgenic PPP1CC2 expression was first detected in early pachytene spermatocytes and remained present through late elongating spermatids (Fig. 2g). The absence of PPP1CC1 immuno-reactivity in brain, testis, and other tissue extracts of transgenic rescue mice confirmed the *Ppp1cc* null background status of these animals (Fig. 2c, d).

**Figure 3:**
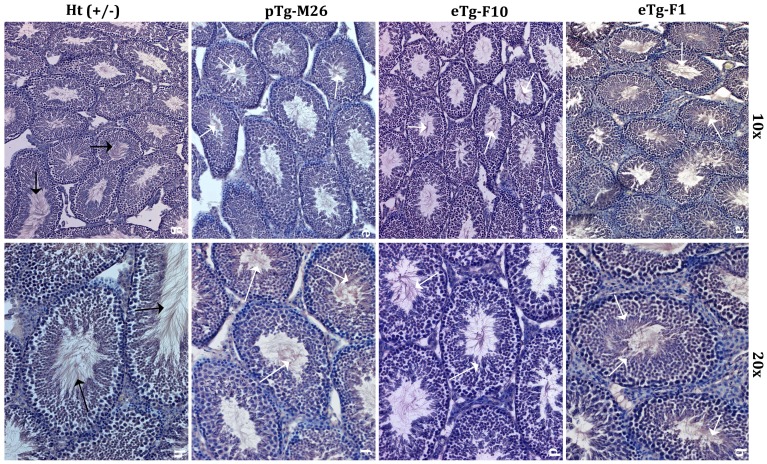
Histology of testis section from rescue animals compared to that of control heterozygous mice. Paraffin embedded adult testis sections from transgenic lines eTg-F1 (**a, b**), eTg-F10 (**c, d**), pTg-M26 (**e, f**) and positive controls (**g, h**) were stained with haematoxylin for comparison of gross tissue architecture. The stained sections were viewed under 10× and 20× magnification using an Olympus 1×70 microscope. Note that both the size and overall architecture of seminiferous tubules of rescue mouse testes were restored in comparison to *Ppp1cc −/−* testis, and were comparable to those of positive control mice. Also notice that the seminiferous tubular lumens are replete with mature testicular spermatozoa, clearly seen in all rescue lines (white arrow) and in positive controls (black arrow). These observations are representative of multiple sections prepared from testes of several animals of each rescue and positive control line.

As shown in Figure 3, morphology of paraffin embedded, haematoxylin stained testis sections from rescue mice appeared normal and comparable to sections from wild-type testis. Unlike those of *Ppp1cc* −/− mice [12], [23], the lumen of the seminiferous tubules of rescue mice were replete with spermatozoa. A full complement of germ cells at various stages of development, including round and early and late elongating spermatids, was also evident. In addition, mean testis weights of mice from all but two rescue lines were significantly higher than the mean testis weight of *Ppp1cc* null mice and comparable to testis weights from *Ppp1cc* +/− controls (Table 1, column 3). Only the pTg-M3 and the eTg-F1 lines had mean testis weights that were significantly different from that of positive controls.

**Table 1:** Comparison of PPP1CC2 levels, testis weight, sperm number and morphology between transgenic rescue lines and control animals.

Transgenic lines	Normalized PPP1CC2 levels compared to Ppp1cc +/− level	Mean testis weight± SEM (in mg)	Mean Sperm number± SEM×107/ml	Sperm Morphology		
				Total number of sperm counted	Normal (%)± SEM	Defective (%)± SEM
*Ppp1cc* +/−	n = 2, 1^c^	n = 10; 114.4±1.9^c^	n = 6; 3.9±0.3^b^	n = 1; 368	95	5^c^
*Ppp1cc −/−*	no expression	n = 6; 68.4±5.4^a^	–	–	–	–
eTg-F10	n = 2; 0.125^a^	n = 3; 110.5±1.9^b,c^	n = 3; 2.1±0.8^a^	n = 2; 323	32±2.8	68±2.8^a,b^
pTg-M3	n = 2; 0.125^a^	n = 2; 79.9±2.9^a,b^	n = 2; 1.4±0.05^a^	n = 2; 981	47±1.2	53±1.2^a,b^
eTg-M7	n = 2; 0.25^a,b^	n = 8; 107.4±4.6^b,c^	n = 5; 1.7±0. 9^a^	n = 2; 566	24±9.9	76±9.9^a^
eTg-F1	n = 5; 0.5^a,b,c^	n = 9; 102.7±2.9^a,b^	n = 5; 2.6±0.5^a,b^	n = 3; 1194	75±0.2	25±0.2^a,b,c^
pTg-M26	n = 3; 0.75^b,c^	n = 5; 108.2±4.2^b,c^	n = 4; 3.7±0.3^a,b^	n = 2; 986	88±2.3	12+2.3^b,c^
pTg-M30	n = 3; 1^c^	n = 4; 108.2±0.5^b,c^	n = 3; 3.7±0.5^a,b^	n = 2; 431	94±1.1	6±1.1^c^

The Kruskal-Wallis non-parametric one-way ANOVA by rank was performed and results were analyzed *post-hoc* by Dunn’s procedure for performing two-tailed multiple pair wise comparisons. Differences were considered significant if *p*<0.05 at a confidence interval of 95%.

**a, b, c…** denote significantly different groups.

**SEM** denotes Standard Error of the Mean.

**n** denotes number of samples/group.

### Increasing PPP1CC2 Steady State Levels in the Testes and Spermatozoa of Rescue Mice Show a Positive Correlation with the Restoration of Fertility

**Figure 4:**
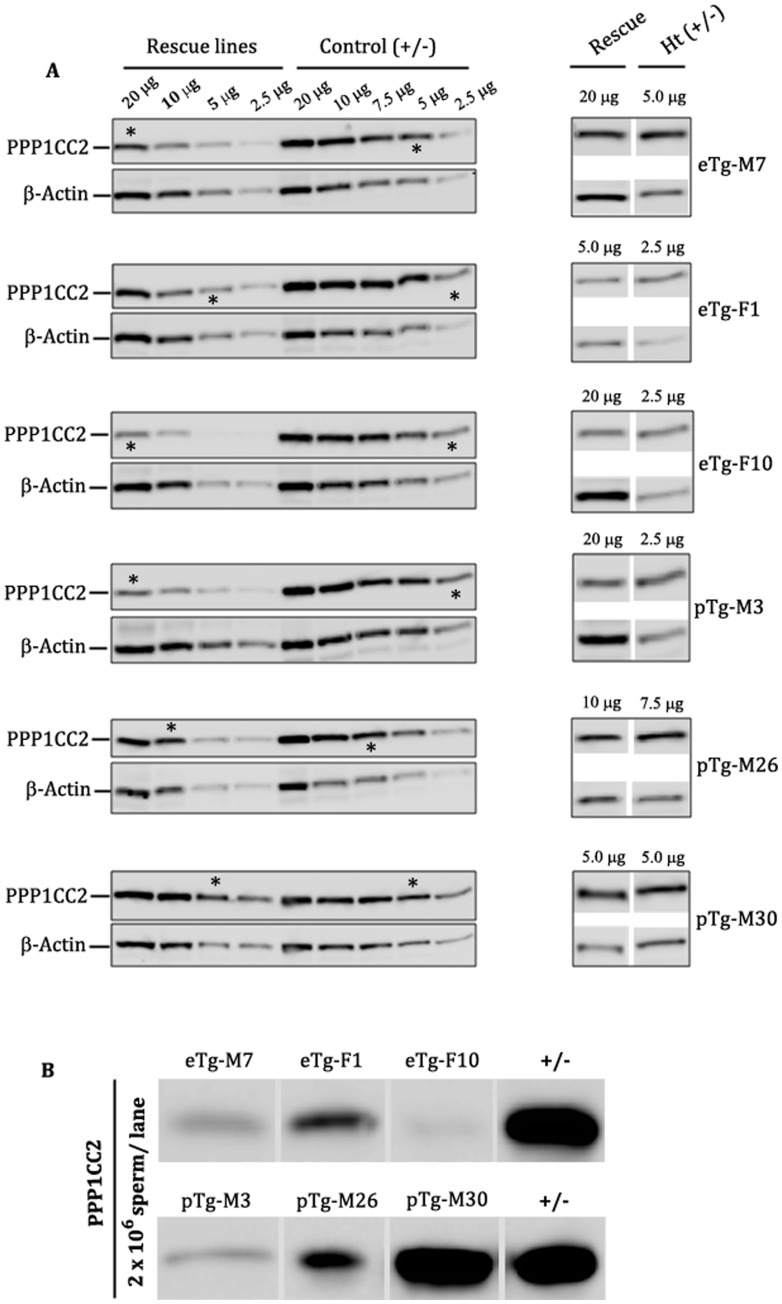
Comparison of steady state levels of transgenically expressed PPP1CC2 in testis and spermatozoa from all transgenic rescue lines. (**A**) Western blot showing the levels of PPP1CC2 expressed in the testis of rescue lines compared with littermate controls. In the left panel protein estimated testis extracts from rescue animals were serially diluted to 20 µg, 10 µg, 5.0 µg and 2.5 µg and 20 µg, 10 µg, 7.5 µg, 5.0 µg and 2.5 µg from positive controls. (*) denotes the lane in which the band corresponding to rescue mouse PPP1CC2 expression is comparable in intensity to a band in a control mouse lane. In the right panels, (*) lanes are shown adjacent to each other for visual comparison. β-Actin was used as loading control. Each blot was repeated with preparations from different animals of the same line to confirm the original data. (**B**) Western blot of whole sperm protein extract demonstrate PPP1CC2 levels in spermatozoa. Protein extract from 2×10^6^ sperm was loaded in each lane for each rescue line and its littermate control. Upper panel represents PPP1CC2 levels in endogenous promoter driven rescue lines (eTg) while the lower panel represents PPP1CC2 levels in the *hPgk2* promoter driven rescue lines (pTg). Note that the PPP1CC2 levels in spermatozoa roughly conform to their levels in the testis.

In order to compare the levels of transgenic PPP1CC2 expressed in the testes of the different rescue lines, protein adjusted amounts of testis extracts from transgenic and control mice were serially diluted and analyzed on western blots (Fig. 4A). Immuno-reactivities in the lanes showing apparent matching intensities within the linear range were adjusted by densitometric analyses (see Materials and Methods). In the endogenous promoter driven lines (eTg-F10, eTg-M7, and eTg-F1) the levels of expressed PPP1CC2 were approximately 12.5%, 25%, and 50%, respectively of levels expressed in the testes of *Ppp1cc+/−* controls (Table 1, column 2). The *hPgk2* promoter lines, pTg-M3, pTg-M26, and pTg-M30, also showed different levels of PPP1CC2 expression (approximately 12.5%, 75%, and 100%, respectively) of positive control expression levels [(Fig. 4A), (Table 1, column 2)]. To further confirm lack of expression in *Ppp1cc −/−* control mice, we compared PPP1CC2 levels in testis across all transgenic lines with that of *Ppp1cc* null animal. *Ppp1cc* null animal are indeed devoid of endogenous expression of PPP1CC2 (see Fig. S1). To assess the levels of PPP1CC2 incorporated into mature spermatozoa, extracts from 2×10^6^ caudal spermatozoa for each transgenic line and *Ppp1cc +/−* controls were compared by western blot analysis. The levels of PPP1CC2 incorporated into mature spermatozoa roughly paralleled testis levels (Fig. 4B).

Males from all six transgenic rescue lines were subsequently tested for fertility as described in Materials and Methods. All males tested from three lines were completely sterile (pTg-M3, eTg-M7 and eTg-F10), and from two lines were completely fertile (pTg-M26 and pTg-M30). However, males from the eTg-F1 line showed variability, with most males exhibiting complete sterility, while others were fertile (Table 2). The mean litter size produced by the fertile eTg-F1 males was slightly, but significantly different from the mean litter size produced by either positive control males or males of the completely fertile pTg-30 line. In addition, the duration between consecutive litters was slightly, but significantly increased for the eTg-F1 males (Table 2). Because of these findings, and because greater than two-thirds of the eTg-F1 males were sterile, the eTg-F1 line was classified as sub-fertile. Together, these results established, for the first time, that absence of PPP1CC1 expression in the mouse testis gives rise to no immediately discernible abnormal phenotype. In addition, since the pTg-M3 line (sterile) and the eTg-F1 line (sub-fertile) had mean testis weights that were significantly different from that of positive controls, while the other sterile lines (eTg-M7 and eTg-F10) had mean testis weights not significantly different from the positive control mean or the means of the other fertile lines, it was reasonable to assume that testis weight had little bearing on the fertility status of rescue mice.

**Table 2:** Fertility Data.

Transgenic lines	Number ofmales testedfor fertility (n)	Number ofmales fertile	Number oflitters	Mean time (days) elapsed between two consecutivelitters ± SEM(min – max)	Average littersize ± SEM	Fertility status
eTg-M7	10	0	0	N/A	N/A	Infertile
eTg-F1	13	4	12	23.1±0.7 (21–27)	7.1±0.6^a^	Sub- fertile
pTg-M26	5	5	11	22.3±0.5 (19–25)	8.6±0.9^a,b^	Fertile
pTg-M30	3	3	9	20.4±0.7 (18–23)	9.2±0.9^b^	Fertile
Control (+/−)	3	3	8	20.9±0.3 (20–21)	9.0±0.7^b^	Fertile

The Kruskal-Wallis non-parametric one-way ANOVA by rank was employed and results were analyzed *post-hoc* by Dunn’s procedure for performing two-tailed multiple pairwise comparisons. Differences were considered significant if *p*<0.05.

**a, b…** denote significantly different groups.

**SEM** denotes Standard Error of the Mean.

### Spermatozoa of Males from Rescue Lines Expressing Transgenic PPP1CC2 Levels below 50% of *Ppp1cc+/−* Levels Exhibit Oligoterato-asthenozoospermia

Statistical analysis (see Materials and Methods) of sperm numbers recovered from the caudae epididymides and ductus deferens of rescue mice showed that sterile lines (pTg-M3, eTg-F10 and eTg-M7) had significantly fewer sperm than *Ppp1cc* +/− positive control mice (p≤0.010, p≤0.025, and p≤0.035, respectively), Table 1 (column 4). In contrast, sperm numbers from line eTg-F1, pTg-M26, and pTg-M30 were lower, but not significantly so, than positive control numbers (p≤0.086, p≤0.525, and p≤0.920, respectively) (Table 1, column 4). These results signified a strong positive correlation between levels of spermiation and the steady state level of PPP1CC2 expression in the testes of rescue mice (Table 1). However, none of the sterile or sub-fertile lines appeared to exhibit severe enough oligospermia to account fully for their reduced ability/inability to fertilize. Thus, we examined other potential causes of infertility in the sterile rescue lines and whether increasing PPP1CC2 expression levels correlated with improved values for the parameters evaluated.

**Figure 5:**
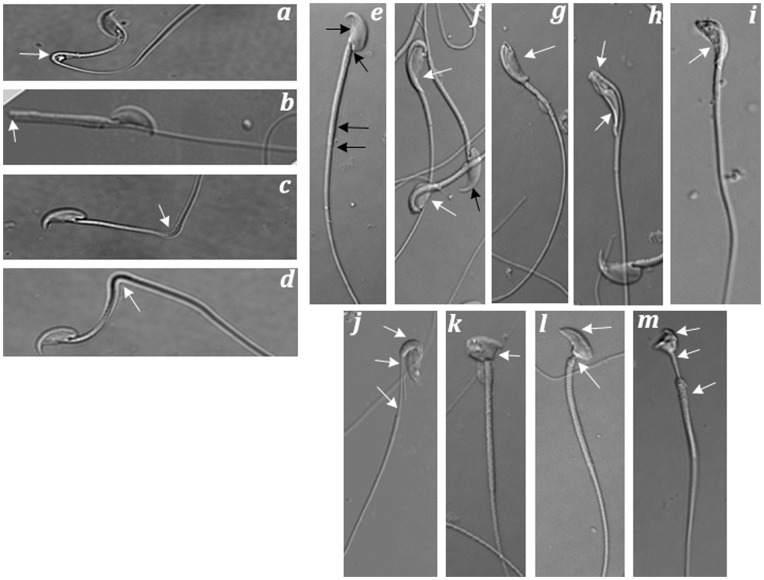
Morphological abnormalities of spermatozoa as seen in rescue lines expressing low levels of PPP1CC2. (**a, b, c, d**) Mature epididymal spermatozoa with aberrant bending between mid-piece and principle piece. The angular variation of the bend ranges from 45° (**a**) to 90° (**c, d**) to 180° (**b**). (**e–i**) A series of frequently observed morphological defect involving jack-knifing of the head between the capitulum and the proximal mid-piece. (**j–m**) Other less common deformities, including shortened mitochondrial sheaths (**j, m**) and malformed heads (**k, l, m**). All micrographs were photographed under DIC optics with an Olympus 70 light microscope. Abnormal regions of the spermatozoa are denoted by white arrows and its corresponding normal regions are denoted by black arrows. These observations are representative of multiple samples from cauda epididymal preparations of different rescue animals.

Sperm gently extruded from the caudae epididymides and vas deferens of rescue mice were fixed in 4% paraformaldehyde for morphological assessment by light microscopy using DIC optics as previously described [24]. Sperm morphology data are shown in Figure 5 and summarized in Table 1 (column 5). Our findings demonstrated that the overwhelming majority of gross morphological abnormalities were, as previously shown [24], defects of the sperm flagellum and connecting piece. More importantly, the proportion of mature sperm with normal morphology was below 50% for rescue mice expressing PPP1CC2 at levels less than 50% of *Ppp1cc +/−* control levels (pTg-M3, eTg-M7, and eTg-F10) (Table 1). As expected, these mice were all from sterile lines. In the sub-fertile line, eTg-F1, and the two fully fertile lines, pTg-M26 and pTg-M30, each expressing PPP1CC2 levels equal to or greater than 50% of positive control levels, the proportion of spermatozoa with abnormal morphology dropped dramatically to approximately 25%, 12%, and 6%, respectively (Table 1). Linear correlation analysis showed a strong positive correlation between increasing steady state levels of testicular PPP1CC2 and increasing percentages of morphologically normal post-testicular spermatozoa.

**Figure 6:**
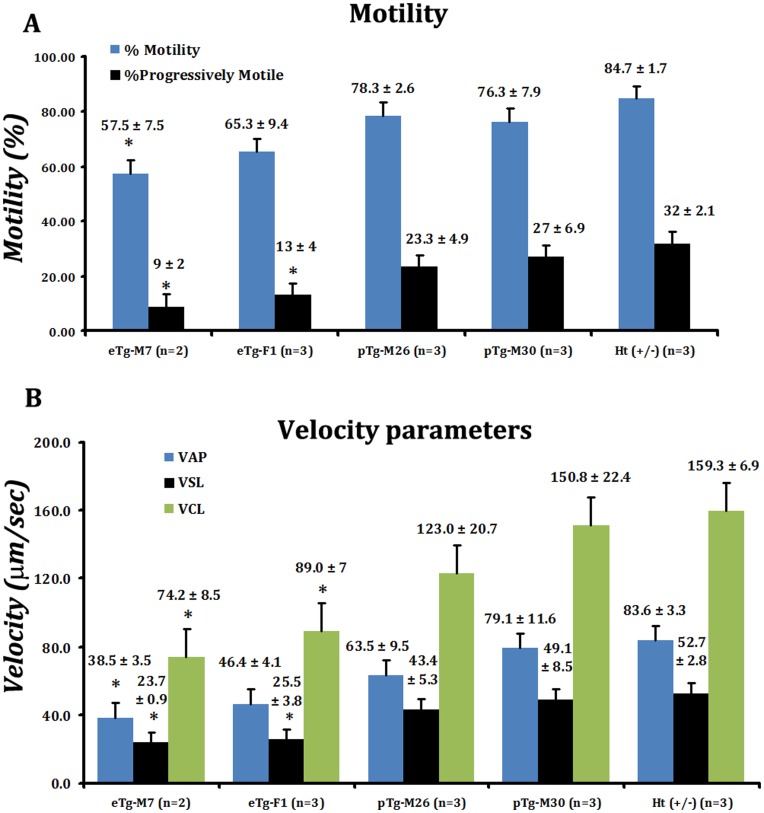
Motility analysis of mature caudal sperm from rescue animals with varying levels of PPP1CC2. Computer assisted sperm analysis of freshly prepared mature cauda epididymal spermatozoa was performed on sperm from adult (8–12 weeks old) mice from different transgenic rescue lines. (**A**) Both total percent motile (blue bars) and progressively motile (black bars) increased as levels of PPP1CC2 increased. Significant differences from positive control mean values of percent motile sperm were observed for males of line eTg-M7, and were observed in lines eTg-M7 and eTg-F1 for percent progressively motile sperm. (**B**) The velocity parameters VAP (average path velocity; blue bars), VSL (straight line velocity; black) and VCL (curvilinear velocity; green bars) generally increased with increasing expression of PPP1CC2. Mean values for VSL and VCL were significantly lower than positive control values in animals with testis levels of PPP1CC2 equal or less than 50% of positive control levels (eTg-M7 and eTg-F1), but mean values for VAP were significantly lower than positive control values only in animals with testis levels of PPP1CC2 less than 50% of positive control levels (eTg-M7). A non-parametric ANOVA by ranks was used for comparison of all groups, followed by post-hoc pairwise analysis by Dunn’s procedure (p<0.05). For each animal ≥10 non-overlapping fields were recorded for analysis. The motility parameters were expressed as mean of n = 3± Standard Error of the Mean (SEM), except for eTg-M7 (n = 2), are shown atop each data bar. ‘*’ denotes motility parameters for which significant differences from positive control values were observed.

Because morphology alone is not always an adequate predictor of fertility [26], we investigated motility parameters of spermatozoa (factors known to be affected by sperm morphology) from rescue mice, and determined whether improving motility values also correlated with increasing expression of transgenic PPP1CC2. Sperm motility parameters were measured as described in Materials and Methods. Both percentages of motile sperm and of progressively motile sperm increased in direct proportion to increasing levels of PPP1CC2 in both testis and sperm of rescue mice. Spermatozoa isolated from mice of the sterile rescue line expressing the highest level of testicular PPP1CC2 of all new sterile lines, eTg-M7, had a significantly lower percentage of motile sperm (57.5±7.5%) than positive controls (84.7±1.7%). Moreover, the percentages of progressively motile sperm recorded from the same line as well as from the sub-fertile line, eTg-F1, were both significantly lower than the mean percentage of progressively motile sperm isolated from positive control males (eTg-M7∶9.0±2.0% and eTg-F1∶13±4.0% compared to ∼32.0±2.1% for positive control sperm). With further increases in levels of PPP1CC2 (lines pTg-M26, pTg-M30) the percentages of motile sperm increased to 78.3±2.6% and 76.3±7.9%, respectively, and the percentages of progressively motile sperm to 23.3±4.9% and 27±6.9%, respectively, both slightly lower than, but not significantly different from control levels (Fig. 6A).

As expected, sperm velocity parameters including average path velocity (VAP), straight line velocity (VSL) and curvilinear velocity (VCL), also increased in proportion to increasing testis levels of PPP1CC2 (Fig. 6B). VSL values recorded for rescue lines eTg-M7 (23.7±0.9 µm/sec) and eTg-F1 (25.5±3.8 µm/sec) were significantly lower than positive control values (52.7±2.8), but VSL improved dramatically for sperm from males for lines showing levels of testicular PPP1CC2 expression greater than 50% of positive control male expression (pTg-M26∶43.4±5.3 µm/sec; and pTg-M30∶49.1±8.5 µm/sec). These latter values were not significantly different from those of positive controls. Measurement of VCL showed a similar trend with the mean values for eTg-M7 (74.2±8.5 µm/sec) and eTg-F1 (89.0±7.0 µm/sec) showing significant deviations from those of positive controls (159.3±6.9 µm/sec). However, VAP values demonstrated a slight deviation from this trend in that the mean value for eTg-F1 sperm was intermediate between the mean values for eTg-M7 and positive control sperm, and not significantly different from either (Fig. 6B).

## Discussion

Testis expression of PP1 isoforms other than PPP1CC2 is confined largely to Sertoli cells, spermatogonia, and pre-leptotene spermatocytes. In contrast, PPP1CC2 expression is restricted predominantly to meiotic and post-meiotic stages of developing male germ cells [12]. Thus, infertility of *Ppp1cc* null males, resulting in the disruption of the spermatogenic process, particularly during mid- and late- spermiogenesis, implies that PPP1CC2 must be critical for the morphological development, spermiation, and fertility of male germ cells [12], [23], [24]. In order to test the proposition that, when appropriately expressed in the testes of *Ppp1cc* null mice, PPP1CC2 would be able to restore their fertility, even in the absence of PPP1CC1 expression, we have expressed transgenic mouse *Ppp1cc2* cDNA, driven either by its endogenous promoter or the testis specific *hPgk2* promoter, in the *Ppp1cc* null background.

The results of these experiments demonstrate spermiogenic roles for PPP1CC2 that are not only isoform specific, but are critical for sperm function in fertilization, while the PPP1CC1 isoform appears to be nonessential for fertility in the presence of levels of PPP1CC2 expression approximating levels expressed by a *Ppp1cc +/−* control male.

Importantly, these studies show that, regardless of promoter choice for transgenic PPP1CC2 expression, restoration of fertility can occur in *Ppp1cc −/−* rescue mice when their testicular PPP1CC2 expression levels cross a lower threshold of at least 50% of the level of PPP1CC2 expression in the testis of *Ppp1cc +/−* control males.

Our findings also found strong positive correlations between the achievement of this threshold and a greater level of transgenic PPP1CC2 expression in the testis of rescue mice and the restoration of expression of traits normally associated with male fertility.

Interestingly, complete fertility was restored only in mice from transgenic lines where PPP1CC2 expression levels were well above 50% of levels in the testis of positive control mice, while males from rescue line eTg-F1, in which the testis expression level of PPP1CC2 reached the lower threshold but did not cross it, were either completely sterile or exhibited sub-fertility. The reason for the stark difference between males of the same line is likely related to differences in genetic background, as rescue lines were generated by two generations of crosses of *Ppp1cc2* transgenic males to outbred CD1 *Ppp1cc −/−* females. Thus, although sterile and sub-fertile males of the eTg-F1 line expressed similar levels of PPP1CC2, and produced a comparable number of epididymal spermatozoa with nearly identical morphological and motility characteristics, their fertility status may have been influenced by subtle epigenetic differences between them.

Nevertheless, regardless of genetic background, we speculate that increasing PPP1CC2 expression by rendering the transgene homozygous in sub-fertile eTG-F1 males or by generating eTg-F1×pTg-M3 (or eTg-F10) doubly heterozygous transgenic males will restore complete fertility to the higher expressing males derived from these crosses.

The requirement for a significant threshold level of PPP1CC2 expression may reflect stoichiometric interactions of the enzyme with its regulators and/or its protein substrates in testis and spermatozoa. It is possible that these interactions target PPP1CC2 to specific structures in developing and mature spermatozoa. A requirement for stoichiometric amounts of the testis specific isoform of the PKA catalytic subunit Cs (Cα2) has also been suggested [27]. It is interesting that a somatic form of the PKA catalytic subunit, Cα1, is expressed in pre-meiotic stages of germ cell development and in Sertoli cells, whereas Cs is expressed in meiotic and post-meiotic developing germ cells, a situation analogous to the expression of the somatically ubiquitous isoform (PPP1CC1) and male germ cell restricted isoform (PPP1CC2) of *Ppp1cc,* respectively [27]. In addition, utilization of an alternate transcription start site providing testis specific Cs with a unique amino terminus replacing the amino terminus of the somatic form [28] is reminiscent of the alternative splicing event that provides PPP1CC2 with a unique, mammalian specific carboxy terminus.

Morphologically defective sperm and male sub-fertility characterize RIα (a PKA regulatory subunit also known as PRKAR1A) +/− mice, possibly due to increased unregulated PKA catalytic activity. Significantly, men with mutations in the PRKAR1A gene have reduced fertility due to defects in sperm morphology and azoo- or oligospermia [29]. While *Ppp1cc +/−* mice are not similarly affected, our findings suggest that further reductions in PPP1CC2 activity in the testis may lead to an increased steady state level of protein phosphorylation, a situation analogous to that which occurs in testis of haploinsufficient RIα +/− mice. Sperm structural abnormalities seen in RIα +/− mice bear striking similarities to those of defective spermatozoa from rescue mice expressing reduced levels of PPP1CC2 [29].

Spermatozoa have little cytoplasm with limited ability of proteins to diffuse within the cell. Consequently each PPP1CC2 catalytic subunit probably has access to a limited number of substrate molecules and therefore may need to be present at a relatively high stoichiometry of enzyme to substrate ratio. It may be noted that lack of PPP1CC isoforms has been associated with gross morphological and ultra structural abnormalities of spermatozoa, including disorganized and supernumerary outer dense fibers, malformed mitochondrial and fibrous sheaths, and defective sperm head morphology [12]. These morphological and ultra-structural deformities were also observed in sperm from the *hPgk2* promoter driven transgenic rat *Ppp1cc2* cDNA rescue experiments where there was very limited expression of PPP1CC2 [24]. Our data now show that it is likely that these sperm morphological defects correlate with either the complete absence or presence of sub-optimal levels of PPP1CC2 in developing germ cells and spermatozoa.

PPP1CC2 has been shown to form large multimeric complexes with Sds22 and I-3, known inhibitory regulators of PP1, in both testis and sperm [30]. Interestingly each of these PP1 regulators, as well as another inhibitory regulator, I-2, appears to have a testis specific isoform produced by an alternative splicing events (manuscript in preparation). It is also noteworthy that both mRNA and protein expression patterns of these testis specific isoforms closely match that of PPP1CC2 in the pubescent and post-pubescent testis.

The requirement for PPP1CC2 among all the PP1 isoforms is surprising given the high degree of homology in the amino acid sequences of the four isoforms (∼90%). The catalytic core of the enzyme is conserved, while the major differences between the isoforms reside in their C-terminal regions. Indeed, PPP1CC2 has a unique ∼22 amino acid carboxy terminal tail, absent from other PP1 isoforms, and it is significant that the carboxy terminal tail is nearly completely conserved in its entirety among all the mammalian species for which annotated genomic databases exist. This indicates functionality specific to PPP1CC2, but to date the functional nature of this carboxy tail has not been completely elucidated.

In summary, we conclude that PPP1CC2 is critical for male fertility. We have shown conclusively that in the absence of PPP1CC1 expression, heterozygous level of PPP1CC2 expression in meiotic and post-meiotic germ cells is necessary for normal spermatogenesis. We thus conclude that normal sperm morphogenesis, effective spermiation, and progressive sperm movement require adequate levels of PPP1CC2 in developing testicular germ cells. This requirement for a threshold level of testis PPP1CC2 for male fertility to occur is a novel observation. We speculate that high levels of PPP1CC2 are required for maintaining stoichiometric interactions with its regulators and substrates in testis and spermatozoa. A potential clinical implication of this study is that both protein and catalytic activity levels of PPP1CC2 could be used as biomarkers for assessment of male fertility.

## Materials and Methods

### Ethics Statement

Transgenic and all other mice used in the present study were housed and used at the Kent State University animal facility. Housing and handling is in accordance with the Kent State animal ethics committee called “Institutional Animal Care and Use Committee (IACUC) under protocol number [268DK 09-09]” approved by the National Institute of Environmental Health Sciences Animal Care and Use Committee.

### Transgene Constructs

#### Endogenous promoter driven transgenic expression of PPP1CC2

The cDNA (including 5′UTR and 3′UTR) of *Ppp1cc2* was PCR amplified after reverse transcription from adult mouse testicular RNA (Omniscript ReverseTranscriptase, Qiagen) using the forward primer 5′ CTC**GAATTC**CATCTTGTTCTTCTCGTG-3′ (introduced EcoRI site shown in bold letters) and the reverse primer 5′-CTC**ATCGAT**AGTCTGAAACCATTCTC-3′ (introduced ClaI site shown in bold letters). The amplified fragment was inserted into a TOPO-TA cloning vector (Invitrogen) for sequence confirmation. The *Ppp1cc2* cDNA fragment released by restriction digestion with EcoRI and ClaI was subcloned into pBluescript SK II (−) vector. An ∼210-bp SV40 poly A signal was PCR amplified from pcDNA4.0 plasmid (forward and reverse primers pairs: 5′-CTC**CTCGAG**TCTCATGCTGGAGTTCT-3′ and 5′-CTC**GGTACC**ACCATGATTACGCCAAG-3′, respectively), and the resultant fragment was subcloned between the XhoI and KpnI sites downstream of the *Ppp1cc2* cDNA. A 3.5-kb genomic region upstream of the *Ppp1cc* gene transcription start site (TSS) was amplified from a genomic BAC clone (Accession# AC127266, Clone**#** RP24-347B12). The primers used for PCR amplification were forward 5′-CTA**GGTACC**CTGGTTGGTTCCTTC-3′ and reverse 5′- CTAT**GAATTC**ATGGCCGCCGACTC-3′ (introduced EcoRI site shown in bold letters). The amplified fragment was subcloned into a TOPO-TA vector (TOPO-3.5-kb-Up) and its sequence was verified. A smaller genomic region spanning 2.6-kb upstream of the transcription start site was amplified from the larger fragment using the forward primer 5′-**GCGGCCGC**ATTGGATTTCAACATTC-3′ (introduced NotI site shown in bold letters) and the same reverse primer. This 2.6kb upstream genomic fragment was subcloned between NotI and EcoRI in the vector containing the *Ppp1cc2* cDNA and the SV40-PolyA tail. The nucleotide sequence of the entire construct was verified before using it for microinjection.

#### Human Pgk2 promoter driven transgenic expression of PPP1CC2

The entire coding sequence for mouse *Ppp1cc2* was amplified by RT-PCR from mouse testicular RNA using a *Pfu* proof reading polymerase (Invitrogen), and was subsequently ligated into the pBluescript SK II (−) vector between the BamHI and XhoI restriction endonuclease sites. Forward and reverse primers were 5′-GT**GGATCC**ATGGCGGATATCGAC-3′ and 5′-CT**CTCGAG**TCACTCGTATAGGAC-3′, respectively. A fragment containing the human *Pgk2* promoter (*hPgk2*) was amplified from the plasmid pCR2.1 (kindly provided by Dr. John McCarrey, University of Texas, San Antonio, USA) was subcloned into the pBluescript SK II (−) backbone between the SacI and XbaI restriction endonuclease sites. Forward and reverse primers used were 5-CTC**GAGCTC**GAGGTTTTTACATATCA-3 and 5-CTC**TCTAGA**GACAATATAAAGACATA-3′, respectively. Finally the SV40 PolyA signal sequence was isolated from the endogenous promoter plasmid (described above) and introduced into the backbone between the XhoI and KpnI sites. All PCR amplifications were carried out using the proofreading *Pfu* polymerase (Invitrogen) and the resulting sequences were verified.

### Generation of Transgenic Mice

The endogenous promoter-*Ppp1cc2* cDNA -SV40 PolyA construct was digested with NotI and KpnI to release a ∼ 4.2-kb fragment. The h*Pgk2*- *Ppp1cc2* -SV40 PolyA construct was digested with KpnI and SacI to release the ∼1.7-kb transgene. The excised fragments were gel purified and micro-injected into the pro-nuclei of fertilized B6SJL eggs, and the injected eggs were implanted into the uteri of pseudopregnant mothers. Both microinjection and embryo implantation were carried out at the Transgenic Facility of Case Western Reserve University (Cleveland, Ohio). Transgenic mouse production and use at Kent State University follows approved institutional animal care and use committee protocols adapted from the National Research Council publication *Guide for the Care and Use of Laboratory Animals*.

### Genotyping and Developing Rescue Lines

Genotyping was performed by PCR with genomic DNA isolated by alkaline lysis of ear punches. The transgene in each line was detected using the 5′- GTGGTTGAAGATGGCTATGA -‘3 (exon 6) forward and 5′- AAGCTGCAATAAACAAGTTGG-3′ (SV40 internal) reverse primer pair. The transgene positive B6SJL founder mice were mated with either, *Ppp1cc−/−* CD1 females or *Ppp1cc*+/− males. Males with genotype *Ppp1cc*+/−; *Tg+* resulting from the above cross were subsequently mated with *Ppp1cc−/−* null females to derive rescue males (*Ppp1cc* −/−; *Tg*+). *Ppp1cc-*null founder mice were originally the gift of Dr. Susan Varmuza, University of Toronto, Toronto, ON).

### Preparation of Mouse Testis and Sperm Protein Extracts for Western Blotting

Adult male mice 8–12 weeks old were used for tissue and sperm extract preparation following methods outlined previously [12], [30].

### Western Blot Analysis

Protein extracts boiled in Laemmli sample buffer and separated by 12% SDS-PAGE were electrophoretically transferred to Immobilon-P PVDF membranes (Millipore Corp., Billerica, MA, USA). After blocking non-specific binding sites with 5% nonfat dry milk in Tris-buffered saline (TTBS: 25 mM Tris-HCl, pH 7.4, 150 mM NaCl, 0.5% Tween-20), blots were incubated with primary antibodies (1∶5000 dilution) overnight at 4°C. The primary antibodies anti-PPP1CC1 and anti-PPP1CC2 were commercially prepared, and their ability to recognize respective isoforms are well documented [12], [24], [30]. After washing, blots were incubated with anti-rabbit/mouse secondary antibody (1∶5000 dilution) conjugated to horseradish peroxidase (GE Healthcare, Piscataway, NJ, USA) for 1 hr at room temperature. Blots were washed with TTBS twice for 15 min each and four times for 5 min each, and then developed with homemade ECL chemiluminescence preparation.

### Morphological Assessment of Spermatozoa from Cauda Epididymal and Vas Deferens

The caudae epididymides and the vas deferens were carefully removed and placed in a petri dish containing 1×PBS. After adipose tissue and blood vessels were removed, tissues were transferred to a new petri dish containing 1ml of 1×PBS. The ductus deferens was squeezed gently with forceps to extrude sperm. The cauda was lightly minced and squeezed gently, and then incubated at 37°C to allow remaining sperm to swim out into the medium. Extruded sperm were collected in a micro- centrifuge tube and kept on ice for 30 min. The tubes were centrifuged at 600×*g* for 5 min to pellet sperm, and the pellet was re-suspended in double distilled H_2_0 (ddH_2_0) and further incubated on ice to inhibit motility. All steps involving pipetting of sperm were performed using large bore pipet tips.

For counting, sperm were diluted (1∶10) in ddH_2_0 and 10 µl of the diluted sperm were loaded onto a Neubauer haematocytometer. For fixation, the sperm pellets were resuspended in freshly prepared 3.75% paraformaldehyde in 1×PBS and incubated on ice for 1 hr. Fixed spermatozoa were mounted on clean slides and sealed under coverslips. Sperm morphology was analyzed under 20x and 60x objectives with a 1×70 Olympus microscope (Melville, NY, USA) using differential interference contrast (DIC) optics.

From each slide, randomly selected fields were observed and the proportion of defective sperm was counted using a cell counter. Spermatozoa with the following morphological characteristics were counted as defective: head jack-knifed at the connecting piece (Fig. 4, e-i) or tail with 45°−180° hairpin bend at the midpiece-principal piece junction (Fig. 4, a-d), malformed heads, and shortened mitochondrial sheath (Fig. 4, j–m).

### Histology and Immunohistochemistry

After removal, testes were immediately fixed in freshly prepared 4% paraformaldehyde in 1×PBS for 12–16 hrs. Fixed tissues were dehydrated by washing in a graded series of ethanol (70%, 95%, and 100%) for 45 mins each, and then permeabilized in CitriSolv (Fischer Scientific) for 30 min. Tissues were then embedded in paraffin and 8-µm thick sections were transferred to poly L-lysine coated slides. For haematoxylin staining and IHC, testis sections were de-paraffinized in Citrisolv and rehydrated by washing in a graded series of decreasing ethanol concentration (100%, 95%, 80% and 70%) as previously reported [12], [30]. Deparaffinized, rehydrated sections were stained for 2 min with haematoxylin stain (Sigma-Aldrich, St. Louis, MO, USA). Slides were washed with distilled water twice for 2 min followed by brief immersion in freshly prepared acid rinse solution (1 ml glacial acetic acid in 49 ml distilled water) 10 times, then transferred to bluing solution (0.1% NaHCO_3_) for one minute, and briefly immersed in fresh distilled water 10 times. Sections were dehydrated through a single change of 70%, 90% and 100% ethanol each and finally immersed in Citrisolv for one minute before mounting**.** Citrate Buffer (10 mM Citric acid, 0.05% Tween20, pH 6.0) was used for antigen retrieval of de-paraffinized and rehydrated testis sections, followed by incubation in blocking media (10% goat serum, 5% BSA in 1×PBS) for 1 hr at room temperature. Sections were incubated with aforementioned anti-PPP1CC1 and anti-PPP1CC2 primary antibodies at 1∶250 dilutions at 4°C overnight followed by three brief washes in 1×PBS. Negative control sections were incubated in blocking solution instead of primary antibody. Sections were subsequently incubated with corresponding Cy3-conjugated secondary antibodies for 1 hr at room temperature. Finally sections were washed several times for 10 min each time in 1×PBS and mounted with Vectashield (Vector Laboratories, Burlingame, CA, USA) mounting media, then examined using a Fluo View 500 Confocal Fluorescence Microscope (Olympus, Melville, NY, USA).

### Sperm Motility Analysis

Spermatozoa were allowed to swim out of lightly minced caudae epididymides into pre-warmed modified human tubal fluid medium, m-HTF (Irwine Scientific) without HEPES, supplemented with 5 mg/ml BSA, as previously described [31]. Briefly, sperm were incubated for 10 min at 37°C in 5% CO_2_ to allow them to swim out and disperse into the media. At the end of 10 min, the petri dish containing sperm was swirled gently to allow further dispersion before measurement of motility parameters. Sperm were diluted 1∶10 in m-HTF medium (representing ∼ 2×10^6^ sperm/ml) and from the diluted sperm suspension 25-µl was loaded onto one chamber of a 100-µm Leja Chamber 2 slide, prewarmed to 37°C using a MiniTherm stage warmer. Large bore pipet tips were used for pipetting sperm suspensions. Sperm motility analysis was performed using CASA (Computer Assisted Sperm Analyzer) equipped with the CEROS sperm analysis system (software version 12.3, Hamilton Thorne Biosciences, Beverly, MA). Motility was analyzed using the default Mouse-2 settings from Hamilton Thorne with minor adjustments [31]. The settings for analysis were: 60frames/sec, 90 frames acquired, minimum contrast: 30, default cell size: 13 pixels, minimum cell size: 4 pixels, default cell intensity: 75, cells progressive if VAP>50 µm/sec and STR >50%, slow cells were counted as motile, low VAP cut off: 10 µm/sec, low VSL cutoff = 0 µm/sec. For each chamber, 10–12 random non-overlapping fields were recorded for analysis. For each transgenic rescue and control line, motility was recorded independently from three animals, n = 3 (8 weeks or older) and the values for each motility parameter were expressed as mean of the three ±SEM, except for eTg-M7 line where n = 2. For the later we could use only two adult males for motility analysis before the line became extinct.

### Protein Concentration Estimation and Densitometry Analysis

Protein concentration of samples was estimated by the Bradford method using a DC Protein Assay Kit (Bio-Rad, USA). Each sample (including standards) was measured in triplicate and the resulting mean absorbance was used for developing a standard curve and subsequent protein concentration determination. To perform quantitative western blot, protein estimated testis extracts from rescue and control animals were serially diluted within the range from 20 µg to 2.5 µg such that the intensity of the band in the blot is linear with the amount of total PPP1CC2 protein in them. To quantify and compare intensities of protein bands between rescue and control animals, Multi Gauge-ver3.X (from Fujifilm Inc) image analysis software was used for densitometry. Bands that appeared to be of equal intensities by visual inspection were selected for densitometry analysis. Densitometry by image analysis of actin bands was used to control for equal loading.

### Statistics

Statistical analyses were performed using the non-parametric Kruskal-Wallis one-way ANOVA by rank. Results were analyzed post-hoc for pairwise significant differences using Dunn’s procedure. All analyses were performed using XLSTAT-Pro software. In all cases differences between samples were considered significant if p≤0.05. Simple linear correlation/regression analyses were performed for all data sets using steady state levels of PPP1CC2 as the independent variable. Levels of correlation were determined with the on-line program, VassarStats (http://faculty.vassar.edu/lowry/VassarStats.html).

## Supporting Information

Figure S1 **Western blot comparing PPP1CC2 levels across different transgenic lines to that of control animals.** Approximately 20 ug of whole testis extracts corresponding to each sample were loaded on to each lane (upper panel). To be noted, *Ppp1cc−/−* animals do not show any detectable levels of PPP1CC2 confirming our earlier observation. The blot was reprobed with β-Actin in the lower panel.
(TIF)
