# Supplementary figures and images for: Significant Expression Levels of Transgenic PPP1CC2 in Testis and Sperm Are Required to Overcome the Male Infertility Phenotype of Ppp1cc Null Mice
Source: PLoS One. 2012 Oct 17;7(10):e47623. doi: 10.1371/journal.pone.0047623 (PMC3474748; doi:10.1371/journal.pone.0047623)

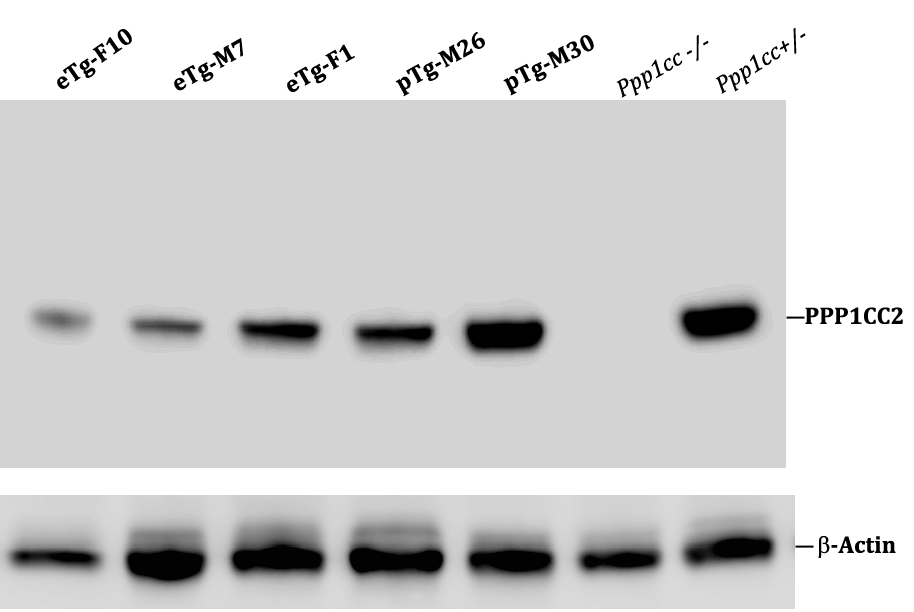

Supplement: Figure S1 — Western blot comparing PPP1CC2 levels across different transgenic lines to that of control animals. Approximately 20 ug of whole testis extracts corresponding to each sample were loaded on to each lane (upper panel). To be noted, Ppp1cc−/− animals do not show any detectable levels of PPP1CC2 confirming our earlier observation. The blot was reprobed with β-Actin in the lower panel. (TIF) [file pone.0047623.s001.tif]
